# Effects of physical exercise intervention on romantic self-perception, anxiety, and psychological wellbeing among female college students

**DOI:** 10.3389/fpsyg.2026.1743525

**Published:** 2026-05-28

**Authors:** Dingwu Liu, Chuang Li, Guanhua Zhu

**Affiliations:** 1 School of Physical Education and Sport Science, QuFu Normal University, Qufu, Shandong, China; 2 Faculty of Philosophy and Political Science, AL-Farabi Kazakh National University, Almaty, Kazakhstan; 3 Institute for the Development of Sport in China, Beijing Sport University, Beijing, China

**Keywords:** anxiety, female college students, mental health, physical exercise, psychological wellbeing, romantic self-perception

## Abstract

This study examined the effects of a structured physical exercise intervention on romantic self-perception, anxiety, and psychological wellbeing among female college students. A two-arm randomized controlled trial with a pretest-posttest design was conducted with 240 undergraduate students from Chinese universities, who were randomly assigned to an intervention group or a control group (*n* = 120 per group). The intervention lasted 8 weeks and was implemented twice weekly for 90 min per session, while the control group continued their usual campus routines. Romantic self-perception, anxiety, and psychological wellbeing were assessed using Likert-type self-report scales. The scales demonstrated satisfactory reliability and convergent validity, with Cronbach’s *α* values ranging from 0.907 to 0.922, composite reliability values ranging from 0.881 to 0.906, and average variance extracted values ranging from 0.529 to 0.554. Baseline comparisons indicated no significant differences between the two groups on the main study variables. Repeated-measures analyses showed significant time effects and significant group × time interaction effects for romantic self-perception, anxiety, and psychological wellbeing. Compared with the control group, participants in the intervention group showed greater improvement in romantic self-perception and psychological wellbeing, as well as a greater reduction in anxiety. Effect size estimates further indicated that changes in the intervention group were substantially larger than those observed in the control group. Parallel mediation analysis showed that the exercise intervention significantly predicted changes in romantic self-perception and anxiety, but the indirect effects of these variables on psychological wellbeing were not statistically significant, suggesting that the benefits of exercise on wellbeing may operate through multiple concurrent pathways beyond those examined in the present model. Overall, the findings suggest that structured physical exercise may serve as a practical behavioral strategy for improving self-perception, reducing anxiety, and promoting psychological wellbeing among female college students, particularly within East Asian university contexts.

## 1 Introduction

### 1.1 Gender imbalance and women’s psychological experiences in sport

Over the past several decades, sport psychology research has been characterized by a persistent gender imbalance, with women’s psychological experiences and sociocultural positions receiving comparatively limited attention in empirical inquiry. Although numerous studies have documented the benefits of physical activity for health, motivation, and performance, many investigations have relied predominantly on male participants, leaving women’s psychological mechanisms in sport less systematically examined (Sabiston et al., 2019). Compared with men, women often encounter more complex forms of bodily and social evaluation in exercise contexts. They face the dual pressures of gender-role expectations and appearance-related anxiety (Krane et al., 2004). One prominent phenomenon in this regard is social physique anxiety, which refers to concerns about how one’s body is evaluated by others. This form of anxiety is particularly prevalent among adolescent girls and female university students and has been shown to undermine intrinsic motivation and reduce the psychological benefits associated with exercise participation (Cox et al., 2013). Beyond appearance-related concerns, gender differences in emotional regulation may also shape women’s psychological experiences in social and relational contexts. Research suggests that women may exhibit greater emotional reactivity and prolonged affective responses under conditions involving stress, anxiety, or interpersonal evaluation (Nolen-Hoeksema, 2012). These gendered psychological patterns indicate that the meaning of physical activity for women may extend beyond physical health and involve broader processes related to self-perception, emotional regulation, and interpersonal confidence. In recent years, international scholarship has increasingly examined body image and self-esteem among women athletes. However, relatively little empirical attention has been given to romantic self-perception, which reflects individuals’ evaluations of their attractiveness, relational worth, and emotional expressiveness in romantic or interpersonal contexts. From a theoretical standpoint, the bodily experiences associated with physical activity—such as enhanced physical competence, improved body control, and a greater sense of physical mastery—may not only strengthen general self-esteem but also extend to more domain-specific forms of self-evaluation, including how women perceive their attractiveness and relational value in intimate contexts. Physical activity may therefore serve as a behavioral context through which women develop a more positive physical self-concept, which in turn shapes their sense of worth and confidence in romantic relationships.

In Chinese university settings specifically, these dynamics may be further amplified by prevailing gender norms and social expectations. Female students in China are frequently subject to appearance-related social pressures and culturally specific ideals of femininity that shape how they evaluate themselves in relational contexts (Vandenbosch and Eggermont, 2012). Compared with many Western contexts, Chinese sociocultural environments tend to place stronger emphasis on physical presentation and gender-role conformity, creating conditions in which women’s self-evaluations in romantic contexts are particularly susceptible to external appearance-based standards and interpersonal comparison (Tolman et al., 2006). Against this backdrop, participation in regular physical activity may carry particular psychological significance for Chinese female university students, offering a structured context in which bodily experiences and self-evaluative processes interact in ways that influence not only general psychological wellbeing but also the more specific domain of romantic self-perception. Understanding the relationships among physical activity, romantic self-perception, and psychological wellbeing therefore represents an important yet underexplored area in women’s mental health research, particularly within East Asian cultural contexts where the intersection of gender norms, bodily experience, and relational identity warrants dedicated empirical attention.

### 1.2 Romantic self-perception and anxiety in women’s mental health

Romantic self-perception refers to individuals’ appraisal of their attractiveness, emotional expressiveness, and perceived value within romantic relationships. Early studies on personality and relationship dynamics suggest that positive self-perceptions are associated with greater relationship satisfaction and emotional stability, whereas negative self-evaluations tend to be linked to anxiety and relational tension (Barelds, 2005). Subsequent research has emphasized the role of sociocultural influences in shaping women’s romantic self-perception. In many societies, women are encouraged to conform to norms of physical presentation and gender propriety, creating what has been described as “visibility pressure,” which may constrain women’s emotional autonomy and sense of security in intimate relationships (Impett et al., 2006). Adherence to socially defined ideals of femininity may also generate tension between the pursuit of attractiveness and the desire for authentic self-acceptance, and within this process body surveillance and appearance-related anxiety have emerged as important risk factors affecting women’s psychological wellbeing (Tolman et al., 2006). As theoretical perspectives evolved, romantic self-perception began to be examined within broader frameworks linking sexuality and body image. Empirical studies indicate that women who experience concerns regarding appearance and body satisfaction tend to report higher levels of shame and anxiety in romantic relationships and lower levels of emotional and sexual intimacy (Sanchez and Kiefer, 2007). In contemporary social environments, the rapid expansion of digital media has further intensified these dynamics. Social media platforms frequently present idealized representations of beauty and desirability that reinforce prevailing appearance standards and encourage sustained self-monitoring, processes that may heighten evaluative pressure and contribute to insecurity and anxiety in romantic contexts (Vandenbosch and Eggermont, 2012). Taken together, existing literature has gradually shifted from viewing romantic self-perception as a purely individual disposition to understanding it as a construct embedded within broader sociocultural and media environments, with empirical evidence consistently linking body image concerns and appearance-related anxiety to diminished relational confidence and reduced psychological wellbeing among women.

Crucially, the factors most consistently identified as undermining women’s romantic self-perception—namely appearance-related anxiety, negative body evaluation, and diminished sense of physical worth—are also among the psychological outcomes most responsive to physical activity interventions. If body surveillance and appearance-based self-monitoring represent core pathways through which sociocultural pressures erode romantic self-perception, then behavioral strategies capable of improving body-related self-evaluations and reducing anxiety may offer a meaningful route to strengthening relational confidence. Physical exercise, by providing repeated experiences of bodily competence, physical mastery, and improved body satisfaction, may therefore serve as a particularly relevant intervention for women whose romantic self-perception is constrained by appearance-related concerns and interpersonal anxiety. This theoretical connection between exercise-induced changes in physical self-concept and downstream improvements in romantic self-perception represents a central but underexamined proposition in women’s mental health research, and one that the present study aims to address empirically.

### 1.3 Physical exercise as a psychosocial intervention

Physical exercise has increasingly been recognized as an important psychosocial intervention with both physiological and psychological implications. Early theoretical perspectives suggested that bodily experiences associated with physical activity can strengthen self-efficacy, enhance perceptions of competence, and contribute to more positive emotional experiences (Fox, 1997). These processes may support broader improvements in self-perception and emotional functioning. Accumulating empirical research has provided further evidence for the positive relationship between physical activity and mental health. A meta-review by Biddle et al. (2019) reported that regular participation in physical activity contributes to greater emotional stability and social adaptability, with stress reduction serving as a key pathway to improved wellbeing. Longitudinal evidence also suggests that individuals who engage in regular exercise show a lower risk of developing anxiety disorders, highlighting the potential protective role of physical activity in emotional regulation (Schuch et al., 2019). Similarly, research on health promotion indicates that more frequent participation in physical activity is positively associated with life satisfaction, social functioning, and psychological wellbeing (Anokye et al., 2012). From a theoretical perspective, Self-Determination Theory (SDT) provides a useful framework for explaining how physical activity contributes to psychological wellbeing. According to SDT, environments that support the satisfaction of individuals’ needs for autonomy, competence, and relatedness can enhance intrinsic motivation and promote psychological functioning (Ntoumanis et al., 2021). Within exercise contexts, the repeated experience of mastering physical challenges and achieving movement-related goals may satisfy the need for competence, while participation in group-based exercise may fulfill the need for relatedness through social interaction and shared effort. Together, these need-satisfying experiences are theorized to strengthen individuals’ physical self-concept—that is, their evaluative beliefs about their bodily capabilities, appearance, and physical worth—which in turn provides a foundation for broader improvements in self-esteem and psychological functioning (Fox, 1997). Consistent with this perspective, studies have shown that participation in physical activity programs can improve emotional regulation and social adjustment across different populations (Neville et al., 2021).

Beyond general psychological functioning, the improvements in physical self-concept generated through exercise participation may extend into more domain-specific forms of self-evaluation, including how individuals assess their attractiveness and relational value in interpersonal and romantic contexts. According to hierarchical models of self-concept, domain-specific self-perceptions—such as those related to physical appearance and bodily competence—feed upward into broader self-evaluative constructs, including self-esteem and interpersonal confidence (Fox, 1997). For women, whose self-evaluations in romantic contexts are often closely tied to perceptions of physical attractiveness and bodily presentation, improvements in physical self-concept resulting from regular exercise may therefore carry particular relevance for romantic self-perception. Specifically, as women develop greater bodily competence and body satisfaction through sustained physical activity, the appearance-related anxiety and self-monitoring that constrain romantic self-perception may be progressively reduced, allowing a more positive and stable sense of relational self-worth to emerge. This proposed pathway from exercise participation through physical self-concept to romantic self-perception remains theoretically grounded but has received limited direct empirical examination, particularly among female university students navigating appearance-related social pressures during a critical period of identity development. The present study therefore seeks to provide empirical evidence for this theoretical proposition by examining whether a structured exercise intervention produces meaningful changes in romantic self-perception alongside broader improvements in anxiety and psychological wellbeing.

### 1.4 Research objectives and hypotheses

Based on the theoretical framework outlined above, the present study proposes four hypotheses. H1, H2, and H3 concern the direct effects of the exercise intervention on the three primary psychological outcomes. H4 addresses the potential mediating mechanisms through which exercise participation may influence psychological wellbeing.

*H1*: Compared with the control group, participants in the exercise intervention will show greater improvement in romantic self-perception.

*H2*: Compared with the control group, participants in the exercise intervention will show a greater reduction in anxiety.

*H3*: Compared with the control group, participants in the exercise intervention will show greater improvement in psychological wellbeing.

Although H1 through H3 address the direct effects of exercise on each outcome independently, the relationship between exercise participation and psychological wellbeing is unlikely to operate in isolation from other psychological changes produced by the intervention. According to the theoretical pathway outlined in the preceding sections, exercise participation is expected to first strengthen physical self-concept and reduce appearance-related anxiety, changes that are themselves reflected in improvements in romantic self-perception and reductions in general anxiety. These psychological shifts may in turn contribute to broader improvements in psychological wellbeing by enhancing relational confidence and emotional stability. Romantic self-perception and anxiety therefore represent theoretically grounded candidate mediators connecting exercise participation to psychological wellbeing, as both variables capture distinct but complementary dimensions of psychological functioning—self-evaluative and emotional—that are central to women’s mental health during the university years. On this basis, the following hypothesis is proposed:

*H4*: Romantic self-perception and anxiety are hypothesized to mediate the relationship between exercise intervention and psychological wellbeing, such that exercise participation will improve psychological wellbeing partly through its positive effects on romantic self-perception and its reducing effects on anxiety.

## 2 Literature review

### 2.1 Physical exercise and women’s mental health

Physical exercise is widely acknowledged to support mental health and continues to attract interest across psychology and public-health disciplines. Early investigations found that regular activity can ease symptoms of depression and anxiety and, for people with clinical depression, may serve as a useful adjunct to treatment (Craft and Perna, 2004). Accumulating evidence shows that exercise benefits both body and mind, assisting in regulating emotion, improving self-esteem, and relieving psychological stress (Anderson and Shivakumar, 2013). In a meta-analysis, Bartley et al. (2013) reported that aerobic exercise reduces anxiety symptoms to a degree comparable with certain psychotherapeutic approaches. Biddle et al. (2019) reviewed multiple studies and observed that regular physical activity is linked to greater emotional stability and adaptability, particularly among adolescents and women, with continued participation associated with more enduring positive mood. A longitudinal cohort study by Schuch et al. (2019) found that individuals who exercise consistently are less likely to develop anxiety disorders, implying that physical activity may help prevent anxiety over time. Evidence synthesized by Stubbs et al. (2017) likewise showed reduced anxiety and stress-related symptoms across diverse samples, while Wipfli et al. (2008) identified a dose–response pattern in which moderate-to-high intensity exercise yielded stronger effects on emotional regulation. Neurophysiological findings add further context. Mikkelsen et al. (2017) reported that exercise influences mental health through changes in neurotransmitter systems, lower inflammation, and modulation of the hypothalamic–pituitary–adrenal axis, mechanisms that appear to strengthen resilience to stress. Rao et al. (2020) showed that sustained aerobic training can lessen depression and anxiety while improving self-esteem and quality of life, providing psychological support for women under emotional strain. Beyond these well-established outcomes, emerging evidence suggests that the benefits of physical activity extend across multiple dimensions of health behavior. Studies conducted among adolescent populations have reported associations between exercise participation and improved sleep quality (El Oirdi et al., 2024), reduced sedentary behavior (El Oirdi et al., 2023), and healthier overall lifestyle habits (El Oirdi et al., 2021), collectively suggesting that regular physical activity may function as a broad-spectrum behavioral resource that supports psychological and physical health across different domains and age groups.

Overall, current evidence portrays physical exercise as a multidimensional intervention that combines physiological and psychological processes. Among women in particular, participation in regular physical activity is associated with emotional balance, stronger self-esteem, and better psychological wellbeing. While the majority of existing research has focused on general mental health outcomes, these findings collectively provide a conceptual basis for examining whether the psychological benefits of exercise extend to more specific domains of self-evaluation, including how women perceive themselves in romantic and interpersonal contexts. The present study builds on this foundation by investigating whether a structured exercise intervention produces meaningful changes in romantic self-perception alongside reductions in anxiety and improvements in psychological wellbeing among female university students.

### 2.2 Body image, self esteem, and romantic self perception

Research on body image and self-esteem offers a useful framework for examining women’s romantic self-perception, and understanding the theoretical connections among these constructs is essential for clarifying how physical exercise may influence relational self-evaluation. Early psychological studies proposed that body image reflects general mental health and functions as a central element of women’s self-worth and social identity. Avalos et al. (2005) created the Body Appreciation Scale to define body appreciation as a favorable attitude toward one’s appearance and bodily capabilities, showing that this orientation supports acceptance of one’s body and helps maintain emotional balance. Importantly, body appreciation is not merely an aesthetic judgment but a functional psychological resource that shapes how individuals evaluate their competence, worth, and emotional stability across different life domains. Hausenblas and Fallon’s (2006) meta-analysis found that regular physical activity enhances women’s perceptions of their bodies and raises self-evaluations, underscoring the role of exercise in shaping body-related attitudes. Carraça et al. (2011) reported that women who experienced improvements in body image during weight-management programs also demonstrated stronger self-regulation and greater persistence in healthy behavior, suggesting that positive body perception contributes to a broader sense of competence and personal control that extends beyond purely physical domains. The connection between body image and romantic self-perception becomes particularly evident when sociocultural pressures and relational contexts are considered. From a sociocultural perspective, Slater and Tiggemann (2011) observed that adolescent girls are often influenced by gender stereotypes and appearance-based comparisons in sports environments, and that external evaluation and self-objectification can erode self-esteem and intensify dissatisfaction with the body. Meltzer and McNulty (2014) found that women receiving affirmative appearance-related feedback from partners reported higher relationship satisfaction and emotional comfort, whereas negative comments were associated with anxiety and self-doubt. A longitudinal investigation by Meltzer et al. (2014) further showed that women, more than men, tend to connect physical appearance with relational value, indicating that perceptions of the body are closely and specifically tied to romantic self-concept rather than to self-esteem in general.

Extending this line of inquiry, recent research has turned toward the positive functions of body appreciation and the conditions under which body-related self-evaluations can be strengthened. Homan and Tylka (2018) proposed a gratitude-based model in which body appreciation fosters psychological wellbeing through self-compassion and positive emotion while reducing exposure to social comparison. Swami et al. (2018) found that spending time in natural environments is linked to higher body satisfaction and self-esteem, pointing to the importance of situational and behavioral factors in shaping body image. Sabiston et al. (2019) observed that body-related self-conscious emotions among women athletes fluctuate across time, but that positive exercise experiences and supportive peer contexts appear to help sustain body confidence, suggesting that structured physical activity may offer a particularly effective context for cultivating stable and positive body-related self-evaluations. Taken together, these findings suggest a theoretically coherent pathway linking physical activity, body image, and romantic self-perception. Body image functions as an intermediary construct through which exercise-induced changes in physical self-evaluation may translate into shifts in how women perceive their relational worth and attractiveness. When physical activity improves body appreciation and reduces appearance-related anxiety, the resulting changes in physical self-concept are likely to extend into the domain of romantic self-perception, given the well-documented tendency for women to connect bodily experience with relational value. This proposed pathway, however, has not been directly examined in intervention research, and the mechanisms through which exercise-related improvements in body image may strengthen romantic self-perception among female university students remain to be empirically established. The present study addresses this gap by testing whether a structured exercise intervention produces measurable changes in romantic self-perception alongside improvements in psychological wellbeing, with body-related self-evaluation serving as a conceptual bridge between these outcomes.

### 2.3 Self concept and social identity through exercise

Physical exercise goes beyond maintaining physical health; it is also a psychological activity through which people form a sense of self and understand their place in society. Early theories regarded bodily experience as a central factor in the emergence of self-awareness, with exercise improving bodily control and feelings of achievement in ways that strengthen self-efficacy and support the development of self-esteem (Fox, 1997). Salmon (2001) introduced the psychoregulatory model of exercise, explaining that physical activity affects neuroendocrine responses and cognitive appraisal in ways that help reduce anxiety and depression and improve the ability to manage stress. Exercise therefore serves both emotional and psychological functions, helping individuals maintain stability and integrate their sense of self over time. Research in neuroscience has further clarified how exercise affects the brain. Deslandes et al. (2009) found that regular physical activity promotes neuroplasticity and maintains neurotransmitter balance, physiological changes that enhance emotional regulation and self-evaluation and thereby form a biological basis for self-concept development. Bernstein and McNally (2018) reported that consistent exercise supports psychological balance when emotions fluctuate, mainly through improvements in self-regulatory capacity, suggesting that exercise strengthens resilience and contributes to a more coherent and stable sense of identity. Beyond individual mechanisms, exercise also carries social meaning. Within the framework of lifestyle psychiatry, Firth et al. (2020) showed that exercise shapes mental health and social identity through health-related behaviors and interpersonal interaction, helping individuals form a more positive view of themselves in relation to others. Further evidence comes from Gan and Jiang (2024), who observed a negative association between exercise participation and social anxiety among university students, with body image acting as a mediator and self-esteem moderating this link, implying that exercise enhances social trust and belonging by improving body perception and self-evaluation.

For female university students in particular, the processes through which exercise shapes self-concept and social identity may extend beyond general psychological functioning into the more specific domain of romantic self-perception. The self-concept changes associated with exercise participation—including stronger self-efficacy, improved body-related self-evaluation, reduced anxiety, and enhanced social belonging—are not confined to the exercise context itself but are likely to generalize to other interpersonal domains, including intimate relationships. When women develop a more positive and stable sense of physical self through regular exercise, this shift in self-concept may translate into greater confidence and perceived relational worth in romantic contexts, given the well-established connection between physical self-evaluation and romantic self-perception documented in the preceding section. Moreover, the social dimensions of exercise participation, such as the experience of positive peer interaction, shared effort, and interpersonal support within group exercise settings, may further reinforce a sense of social competence and relational confidence that carries over into romantic self-perception. Taken together, the evidence reviewed in this section suggests that exercise functions as an integrated practice connecting emotion regulation, identity development, and social adaptation, and that these processes provide a theoretically grounded basis for expecting that participation in structured physical activity will strengthen romantic self-perception alongside broader improvements in psychological wellbeing among female university students.

### 2.4 Research gaps and conceptual model

Extensive research has shown that physical exercise contributes to emotion regulation, anxiety relief, and social adjustment, yet the psychological pathways linking exercise with romantic self-perception and psychological wellbeing have received far less systematic attention. Most studies describe direct associations between physical activity and mental health, whereas the indirect effects of exercise on emotional identity and relationship-related self-evaluation remain underexplored. Vankim and Nelson (2013) examined college populations and reported that vigorous activity relates closely to mental health, social behavior, and perceived stress, pointing to complex interactions among exercise participation, social involvement, and emotional regulation. Even so, much of this work falls within the field of health-behavior research and seldom addresses how exercise influences emotional self-concept or romantic self-perception specifically. Interruptions in exercise routines have been linked to rising anxiety and emotional instability, suggesting that exercise operates as a psychological resource as well as a health behavior (Weinstein et al., 2017). Physiological evidence further explains its mood-regulating potential, as exercise modulates neurotransmission, reduces inflammation, and enhances brain plasticity—processes that underpin both antidepressant and anxiolytic effects (Kandola et al., 2019). Recent scholarship has extended inquiry into social-psychological contexts. Duan et al. (2022) showed that engagement in public sports activities encourages prosocial behavior through the combined effects of flow experience and subjective wellbeing, illustrating that exercise may promote positive emotion and strengthen social bonds. Neuroscientific studies provide converging evidence, with Zhang et al. (2024) identifying cerebellar activation as a key factor in exercise-related anxiety reduction, indicating that neural systems supporting rapid emotional recovery contribute to psychological stability. Institution-based programs have also revealed the potential benefits of structured physical education for adolescents’ body image and wellbeing, though questions remain about how these programs can be sustained across educational settings (Torres, 2021), and evidence suggests that well-designed exercise curricula may improve both mental wellbeing and performance among young participants (Kaur et al., 2025).

Despite the breadth of evidence reviewed above, a critical gap remains: no integrated conceptual model has been proposed to explain how exercise shapes romantic self-perception and psychological wellbeing through identifiable psychological pathways, particularly among female university students. Drawing on the theoretical perspectives outlined in the preceding sections, the present study proposes a conceptual framework organized around three interconnected propositions. First, participation in structured physical exercise is expected to strengthen physical self-concept by providing repeated experiences of bodily competence, mastery, and body satisfaction, and these improvements in physical self-evaluation are theorized to extend into the domain of romantic self-perception by reducing appearance-related anxiety and enhancing perceived relational worth. Second, exercise participation is expected to reduce general anxiety through both physiological mechanisms—including modulation of neurotransmitter systems and stress-response pathways—and psychological mechanisms involving improved self-regulatory capacity and emotional stability. Third, these changes in romantic self-perception and anxiety are proposed to contribute to broader improvements in psychological wellbeing, as greater relational confidence and reduced emotional distress represent two of the most proximal determinants of positive psychological functioning among women. Together, these three propositions define a parallel mediation model in which exercise participation influences psychological wellbeing both directly and indirectly through its effects on romantic self-perception and anxiety. This framework is specifically grounded in the developmental context of female university students in China, where appearance-related social pressures, gender-role expectations, and the demands of university life create conditions under which the psychological benefits of structured exercise may be particularly pronounced. The present study seeks to provide the first direct empirical test of this conceptual model using a randomized controlled trial design.

## 3 Methods

### 3.1 Study design

This study employed a two-arm randomized controlled trial (RCT) to examine the psychological effects of a structured physical exercise intervention among female university students. The primary objective of the study was to evaluate whether participation in an organized exercise program could improve romantic self-perception and psychological wellbeing while reducing anxiety. Participants were randomly assigned to either an exercise intervention group or a control group. The intervention lasted for 8 weeks, and psychological outcomes were assessed at two time points: baseline (pretest) before the intervention began and posttest immediately after completion of the intervention period. This design allowed the study to compare both within-group changes over time and between-group differences in psychological outcomes. All participants provided written informed consent prior to participation. Participants were informed that their participation was voluntary and that they could withdraw at any time without penalty. It should be noted that the present study employed a passive control condition rather than an active control group. Because participants in the intervention group engaged in structured group-based sessions, it is not possible to fully disentangle the specific effects of physical exercise from the non-specific effects of social interaction, group cohesion, and structured routine participation. Future research should consider incorporating an active control condition—such as a social or recreational group activity matched for contact time—in order to isolate the contribution of exercise per se to the observed psychological outcomes.

### 3.2 Participants and recruitment

Participants were recruited from three universities located in eastern China through campus advertisements, classroom announcements, and online recruitment notices distributed through university communication platforms. Recruitment materials invited female students to participate in a study on physical activity and psychological wellbeing. Interested students completed an online screening questionnaire to determine eligibility. To be included in the study, participants had to be female undergraduate students between 18 and 24 years of age, report no medical conditions that would prevent them from engaging in moderate physical activity, and not currently participate in structured exercise more than twice per week. Individuals were excluded if they reported severe psychological disorders, recent musculoskeletal injuries, or current psychological treatment specifically targeting anxiety-related symptoms. A total of 268 students initially volunteered to participate. After screening for eligibility, 240 participants met all inclusion criteria and were enrolled in the study. Participants were then randomly assigned to either the exercise intervention group (*n* = 120) or the control group (*n* = 120). During the eight-week intervention period, six participants withdrew from the study because of scheduling conflicts or personal reasons, including three from the intervention group and three from the control group. Consequently, the final dataset included 234 participants, with 117 in each group. To reduce bias associated with attrition, analyses followed the intention-to-treat principle. Missing posttest observations resulting from dropout were handled using expectation–maximization estimation in order to preserve the randomized structure of the dataset.

### 3.3 Randomization procedure

Participants were randomly assigned to experimental conditions using a computer-generated block randomization procedure designed to maintain balanced group allocation throughout recruitment. A block size of four was used to ensure similar numbers of participants in the intervention and control groups as enrollment progressed. The randomization sequence was generated by a research assistant who was not involved in recruitment, intervention delivery, or statistical analysis. Group assignments were sealed in sequentially numbered opaque envelopes to maintain allocation concealment. After participants completed the baseline questionnaire, the corresponding envelope was opened and participants were informed of their group assignment. Because of the behavioral nature of the intervention, participants could not be blinded to group assignment. However, research assistants responsible for data management and statistical analysis were not involved in intervention delivery and remained blind to group allocation during data coding and analysis. This procedure was implemented to reduce experimenter bias in the handling of outcome data.

### 3.4 Exercise intervention

Participants assigned to the intervention group took part in a structured group-based exercise program lasting 8 weeks. Exercise sessions were conducted twice per week, and each session lasted approximately 90 min. All sessions were supervised by trained physical education instructors with prior experience in organizing university-level fitness activities. Each session followed a standardized three-phase structure. The first phase consisted of a 10- to 15-min warm-up designed to gradually increase heart rate and prepare the body for physical activity through dynamic stretching and light aerobic movements. The second phase included approximately 60 min of moderate-intensity aerobic and functional exercise, including rhythmic aerobic routines, light resistance exercise using body weight, and flexibility training. Exercise intensity was maintained at a moderate level corresponding to approximately 60 to 70% of estimated maximum heart rate. The final phase involved a 10- to 15-min cool-down period consisting of stretching and relaxation exercises. The program was designed to promote regular physical engagement while maintaining a supportive and non-competitive environment. Attendance was recorded at each session to monitor adherence. Participants attended an average of 14.3 out of 16 sessions, indicating a high level of compliance. No exercise-related injuries were reported during the intervention period.

### 3.5 Control condition

Participants in the control group did not participate in the structured exercise program and were asked to maintain their usual daily routines during the eight-week study period. They continued their normal academic and recreational activities without receiving any organized physical activity sessions from the research team. To reduce differential attrition and maintain engagement, control group participants were informed that optional exercise workshops would be offered after the study had concluded. This procedure was intended to maintain participation while avoiding the introduction of additional activities that might influence the psychological outcomes. However, it is acknowledged that the absence of an active control condition limits the extent to which the observed intervention effects can be attributed solely to the physical exercise component. Factors such as the Hawthorne effect, increased social interaction, and the psychological benefits of structured routine participation may have contributed to the improvements observed in the intervention group. This limitation is discussed further in the limitations section.

### 3.6 Measures

All psychological variables were measured using self-report questionnaires administered at baseline and posttest through a secure online survey platform. All scales used a five-point Likert response format ranging from 1 (strongly disagree) to 5 (strongly agree), with higher scores indicating higher levels of the measured construct unless otherwise specified. Romantic self-perception (RSP) was assessed using a six-item scale developed for this study based on prior research on relational self-evaluation and romantic self-perception in intimate relationships (Murray et al., 1996). The scale development procedure involved generating an initial item pool based on the theoretical definitions of romantic self-perception proposed in the existing literature, followed by expert review by three researchers with expertise in social psychology and relationship research to evaluate content relevance and representativeness. Items were retained or revised based on expert consensus, resulting in a final six-item scale. The scale captures individuals’ perceptions of their attractiveness, relational desirability, and confidence in potential romantic interactions. Example items include “I feel confident about my attractiveness in romantic contexts” and “I believe I am capable of forming meaningful romantic relationships.” Anxiety (ANX) was measured using a seven-item scale adapted from widely used measures of general anxiety symptoms in psychological research, drawing conceptually on the framework of the Generalized Anxiety Disorder scale (Spitzer et al., 2006). The items assessed experiences of nervousness, worry, and difficulty relaxing in everyday and interpersonal situations; example items include “I felt nervous or anxious in social situations” and “I found it difficult to relax.” Psychological wellbeing (WBE) was assessed using an eight-item scale adapted from established measures of positive psychological functioning and life satisfaction grounded in the theoretical framework of psychological wellbeing proposed by Ryff and Keyes (1995). The scale captures individuals’ sense of life satisfaction, emotional balance, vitality, and optimism about the future, with example items including “I feel satisfied with my life” and “I feel optimistic about my future.” All scales were translated and adapted using a translation–back translation procedure to ensure linguistic accuracy (Brislin, 1970). Two bilingual researchers independently translated the original items into Chinese, and a third bilingual expert translated them back into English. Discrepancies were discussed and resolved to ensure conceptual equivalence. The psychometric properties of the scales were evaluated using Cronbach’s alpha, composite reliability (CR), and average variance extracted (AVE), and the detailed reliability and validity statistics are reported in the Results section. Discriminant validity was further supported using the Fornell-Larcker criterion, whereby the square root of the AVE for each construct exceeded the maximum inter-construct correlation, confirming that each construct was more strongly related to its own indicators than to those of other constructs in the model. To reduce the potential influence of common method bias arising from the use of self-report measures collected at the same time points, several procedural remedies were implemented. Participants were assured of the anonymity of their responses, informed that there were no correct or incorrect answers, and instructed to respond as honestly as possible. Additionally, the questionnaire was structured so that scales measuring different constructs were separated by brief instructional pages to reduce the salience of inter-construct associations during completion.

### 3.7 Control variables

Several demographic and behavioral variables were included as control variables in order to account for potential confounding influences. Age was measured in years based on self-reported participant information. Body mass index (BMI) was calculated from self-reported height and weight. Participants also reported their current romantic relationship status (0 = single, 1 = in a relationship). In addition, prior exercise habits were measured by asking participants to report the average number of times per week they engaged in physical exercise before the intervention. These variables were included in the mediation analysis to control for individual differences that might influence changes in psychological wellbeing (see Table 1).

**Table 1:** Indicator system of the effects of physical exercise intervention on romantic self perception and psychological well being among female college students.

Variable	Definition	Items	Example content	Scale	Direction
Exercise intervention	Participation in the 8-week structured exercise program	–	Group assignment (0 = control, 1 = intervention)	Binary	Higher = intervention exposure
Romantic self-perception (RSP)	Positive evaluation of one’s attractiveness, relational worth, and confidence in romantic contexts	6	attractiveness, relationship confidence, relational worth	5-point Likert	Higher = more positive RSP
Anxiety	Self-evaluative and generalized anxious affect in daily and interpersonal contexts	7	tension, worry, nervousness, difficulty relaxing	5-point Likert	Higher = higher anxiety
Psychological wellbeing (WBE)	Positive psychological functioning reflected in vitality, satisfaction, optimism, and emotional balance	8	life satisfaction, vitality, emotional balance	5-point Likert	Higher = better wellbeing
Age	Participant age in years	1	self-reported age	Continuous	–
BMI	Body mass index	1	weight/height^2^	Continuous	–
Relationship status	Current romantic relationship status	1	0 = single, 1 = in a relationship	Binary	–
Prior Exercise habit	Average exercise frequency before intervention	1	times per week	Continuous	Higher = more prior exercise

### 3.8 Statistical analysis

All statistical analyses were conducted using Python (version 3.13.9) with commonly used statistical libraries, including pandas, numpy, scipy, and statsmodels. Descriptive statistics were first calculated to examine the distributions of the main study variables. Normality of the variables was assessed using the Shapiro–Wilk test. Prior to conducting the repeated-measures analyses, the assumption of homogeneity of variance was evaluated using Levene’s test, and results indicated no significant violations across the study variables (all ps > 0.05). The assumption of sphericity was not applicable in the present study given that the time factor contained only two levels (pretest and posttest). Although several Shapiro–Wilk tests yielded marginally significant results, repeated-measures ANOVA is generally considered robust to mild departures from normality, particularly with sample sizes comparable to those of the present study. Reliability and validity of the measurement scales were evaluated using Cronbach’s alpha, composite reliability (CR), and average variance extracted (AVE). Baseline equivalence between the intervention and control groups was tested using independent-samples t-tests for the primary continuous study variables. To evaluate intervention effects on romantic self-perception, anxiety, and psychological wellbeing, two-way repeated-measures analysis of variance (ANOVA) was conducted to examine the main effects of group, time, and their interaction. This analysis allowed the study to determine whether changes from pretest to posttest differed between the intervention and control groups. Effect sizes were calculated using Cohen’s d for within-group pre–post changes and between-group change differences. To further examine the psychological mechanisms underlying the intervention effects, a parallel mediation analysis was conducted using a bootstrapping procedure with 5,000 resamples. In this model, exercise group assignment served as the independent variable, changes in romantic self-perception and anxiety were specified as parallel mediators, and change in psychological wellbeing served as the outcome variable. Age, BMI, relationship status, prior exercise habit, and baseline psychological wellbeing were included as control variables. Indirect effects were considered statistically significant when the 95% bootstrap confidence intervals did not include zero.

## 4 Results

### 4.1 Descriptive statistics and normality test

Table 2 presents the descriptive statistics and Shapiro–Wilk normality test results for romantic self-perception (RSP), anxiety (ANX), and psychological wellbeing (WBE) across the control and intervention groups at both pretest and posttest stages. The normality tests indicated that most variables did not significantly deviate from normal distributions (*p* > 0.05), although a few cases showed marginal departures. These marginal departures were considered unlikely to threaten the validity of subsequent parametric analyses, given that repeated-measures ANOVA is generally robust to mild violations of normality when sample sizes are adequate, as is the case in the present study (*n* = 234). Overall, the distributions were considered acceptable for parametric analyses. At baseline, the two groups showed very similar mean levels across all three variables. The control group reported a mean RSP score of 3.08 (SD = 0.66), while the intervention group reported a mean of 3.09 (SD = 0.56). Baseline anxiety levels were also comparable between groups (Control: *M* = 2.98, SD = 0.59; Intervention: *M* = 2.99, SD = 0.65). Psychological wellbeing showed identical mean values at pretest in both groups (*M* = 3.11), with standard deviations of 0.64 and 0.56 for the control and intervention groups, respectively. At posttest, the control group displayed only small changes across variables, with RSP increasing slightly to 3.18, ANX decreasing to 2.94, and WBE remaining at 3.11. In contrast, the intervention group showed larger numerical changes over time. The mean RSP score increased from 3.09 at pretest to 3.45 at posttest, while the mean ANX score decreased from 2.99 to 2.71. Psychological wellbeing in the intervention group increased from 3.11 at baseline to 3.48 at posttest. These descriptive results indicate that the intervention group experienced larger mean changes across the measured variables compared with the control group.

**Table 2:** Results of descriptive statistics and tests of normality.

Variable	Group	Time	*M*	SD	Shapiro–Wilk (p)
RSP	Control	Pre	3.08	0.66	0.366
RSP	Control	Post	3.18	0.62	0.049
RSP	Intervention	Pre	3.09	0.56	0.093
RSP	Intervention	Post	3.45	0.59	0.327
ANX	Control	Pre	2.98	0.59	0.013
ANX	Control	Post	2.94	0.64	0.106
ANX	Intervention	Pre	2.99	0.65	0.238
ANX	Intervention	Post	2.71	0.67	0.229
WBE	Control	Pre	3.11	0.64	0.241
WBE	Control	Post	3.11	0.43	0.040
WBE	Intervention	Pre	3.11	0.56	0.197
WBE	Intervention	Post	3.48	0.37	0.207

M, mean; SD, standard deviation.

### 4.2 Reliability and validity analysis

Table 3 reports the reliability and validity indicators for the three psychological constructs included in the study: Romantic Self-Perception (RSP), Anxiety (ANX), and Psychological WellBeing (WBE). All scales demonstrated satisfactory levels of internal consistency. Cronbach’s *α* values ranged from 0.907 to 0.922, exceeding the commonly accepted threshold of 0.70 and indicating strong internal reliability. Composite reliability (CR) values were also high, ranging from 0.881 to 0.906, suggesting that the observed indicators consistently represented their respective latent constructs. In addition, the average variance extracted (AVE) values for all three scales were above the recommended cutoff of 0.50 (RSP = 0.554, ANX = 0.529, WBE = 0.546), indicating adequate convergent validity. The maximum correlation among latent variables was 0.179, which is substantially lower than the AVE values of each construct, supporting discriminant validity among the measured variables. Taken together, these results indicate that the scales demonstrated satisfactory internal consistency, convergent validity, and discriminant validity, and were suitable for subsequent statistical analyses. To assess discriminant validity using the Fornell-Larcker criterion, the square root of the AVE was calculated for each construct: RSP = 0.744, ANX = 0.727, and WBE = 0.739. Each of these values exceeded the maximum inter-construct correlation of 0.179, confirming that each construct shared more variance with its own indicators than with those of any other construct in the model.

**Table 3:** Comprehensive analysis of reliability and validity results.

Scale	No. of dimensions	Cronbach’s α	Composite reliability (CR)	Average variance extracted (AVE)	Maximum correlation
Romantic self-perception (RSP)	1	0.907	0.881	0.554	0.146
Anxiety (ANX)	1	0.909	0.887	0.529	0.179
Psychological wellbeing (WBE)	1	0.922	0.906	0.546	0.179

All scales demonstrated satisfactory internal consistency and convergent validity.

### 4.3 Baseline equivalence between the intervention and control groups

Table 4 reports the independent-samples t-test results comparing the baseline levels of romantic self-perception (RSP), anxiety (ANX), and psychological wellbeing (WBE) between the control and intervention groups prior to the start of the intervention. The results indicate that no statistically significant differences were observed between the two groups at baseline. For romantic self-perception, the control group reported a mean score of 3.07 (SD = 0.66), while the intervention group reported a mean of 3.08 (SD = 0.56), with the difference not reaching statistical significance (*t* = −0.12, *p* = 0.903). Baseline anxiety levels were also comparable between the two groups, with means of 2.97 (SD = 0.60) in the control group and 3.00 (SD = 0.64) in the intervention group (t = −0.30, *p* = 0.766). Similarly, psychological wellbeing showed nearly identical baseline levels across groups, with mean scores of 3.12 (SD = 0.64) for the control group and 3.11 (SD = 0.56) for the intervention group (*t* = 0.13, *p* = 0.893). These results indicate that the two groups were statistically comparable on the primary psychological variables before the intervention began, suggesting that the randomization procedure successfully produced balanced baseline conditions for the subsequent analyses.

**Table 4:** Independent samples *t*-test results for baseline comparisons.

Variable	Control M (SD)	Intervention M (SD)	*t*	*p*
RSP	3.07 (0.66)	3.08 (0.56)	−0.12	0.903
ANX	2.97 (0.60)	3.00 (0.64)	−0.30	0.766
WBE	3.12 (0.64)	3.11 (0.56)	0.13	0.893

No significant baseline differences were found between the intervention and control groups.

### 4.4 Two-way repeated measures ANOVA results

**Figure 1:**
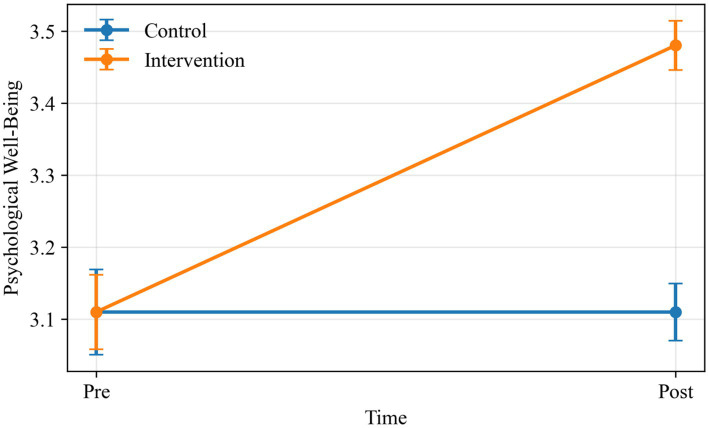
Group × time interaction on psychological wellbeing.

Table 5 presents the results of the two-way repeated-measures ANOVA examining the effects of group (intervention vs. control), time (pretest vs. posttest), and their interaction on romantic self-perception (RSP), anxiety (ANX), and psychological wellbeing (WBE). For RSP, the main effect of time was statistically significant (*F* = 115.28, *p* < 0.001, partial η^2^ = 0.332), indicating an overall change between the pretest and posttest measurements. The main effect of group was not significant (*F* = 2.05, *p* = 0.154). However, a significant group × time interaction was observed (*F* = 36.30, *p* < 0.001, partial η^2^ = 0.135), indicating that the magnitude of change differed between the intervention and control groups. A similar pattern was observed for anxiety. The main effect of time was significant (*F* = 70.28, *p* < 0.001, partial η^2^ = 0.232), whereas the main effect of group was not significant (*F* = 0.20, *p* = 0.653). The interaction between group and time was statistically significant (*F* = 36.96, *p* < 0.001, partial η^2^ = 0.137), suggesting differential changes across groups over time. For psychological wellbeing, the main effect of time was also significant (*F* = 46.83, *p* < 0.001, partial η^2^ = 0.168), while the main effect of group was not significant (*F* = 0.25, *p* = 0.615). The group × time interaction reached statistical significance (*F* = 46.83, *p* < 0.001, partial η^2^ = 0.168). As illustrated in Figure 1, the intervention group showed larger mean changes between the pretest and posttest measurements compared with the control group. These interaction effects indicate that the trajectories of change across time differed between the two groups.

**Table 5:** Two-way repeated measures analysis of variance.

DV	Effect	*F*	*p*	Partial η^2^
RSP	Group	2.05	0.154	0.009
RSP	Time	115.28	< 0.001	0.332
RSP	Group × Time	36.30	< 0.001	0.135
ANX	Group	0.20	0.653	0.001
ANX	Time	70.28	< 0.001	0.232
ANX	Group × Time	36.96	< 0.001	0.137
WBE	Group	0.25	0.615	0.001
WBE	Time	46.83	< 0.001	0.168
WBE	Group × Time	46.83	< 0.001	0.168

Significant Group × Time interactions were observed for all three outcomes, indicating that the intervention group showed greater changes over time than the control group.

### 4.5 Effect size analysis

Table 6 reports the effect size estimates (Cohen’s d) for changes observed in romantic self-perception (RSP), anxiety (ANX), and psychological wellbeing (WBE). For the intervention group, large effect sizes were observed for all three variables. The effect size for RSP was d = 1.13, indicating a substantial increase from pretest to posttest. Anxiety showed a negative effect size (*d* = −0.95), reflecting a meaningful decrease in anxiety levels following the intervention. Psychological wellbeing also demonstrated a large positive effect size (*d* = 0.93). According to conventional benchmarks proposed by Cohen (1988), effect sizes of d ≥ 0.80 are classified as large. All three within-group effect sizes for the intervention group exceeded this threshold, indicating that the observed changes were not only statistically significant but also practically meaningful in magnitude. In contrast, the control group exhibited considerably smaller changes. The effect size for RSP in the control group was *d* = 0.30, while anxiety showed a small decrease (*d* = −0.15). Psychological wellbeing in the control group showed an effect size of *d* = 0.00, indicating a complete absence of change over the same eight-week period. This contrast between the intervention and control groups provides additional evidence that the observed improvements in the intervention group are unlikely to reflect spontaneous fluctuation or regression to the mean. When comparing the magnitude of change between groups, the between-group effect sizes were moderate to large for all variables. The between-group change effect size was *d* = 0.79 for RSP, *d* = −0.79 for anxiety, and *d* = 0.89 for psychological wellbeing. These between-group effect sizes approach or exceed the conventional threshold for large effects, suggesting that the differences in psychological change between the intervention and control groups were substantial enough to be of practical and clinical significance, beyond what would be expected from statistical significance alone. In educational and health promotion contexts, effect sizes of this magnitude are generally considered sufficient to justify the implementation of structured exercise programs as a meaningful behavioral strategy for improving psychological outcomes among university students.

**Table 6:** Effect size results (Cohen’s d).

Variable	Group	Cohen’s d
RSP	Intervention	1.13
RSP	Control	0.30
RSP	Between-group change	0.79
ANX	Intervention	−0.95
ANX	Control	−0.15
ANX	Between-group change	−0.79
WBE	Intervention	0.93
WBE	Control	0.00
WBE	Between-group change	0.89

Negative values for ANX indicate a reduction in anxiety after the intervention.

### 4.6 Parallel mediation analysis

**Figure 2:**
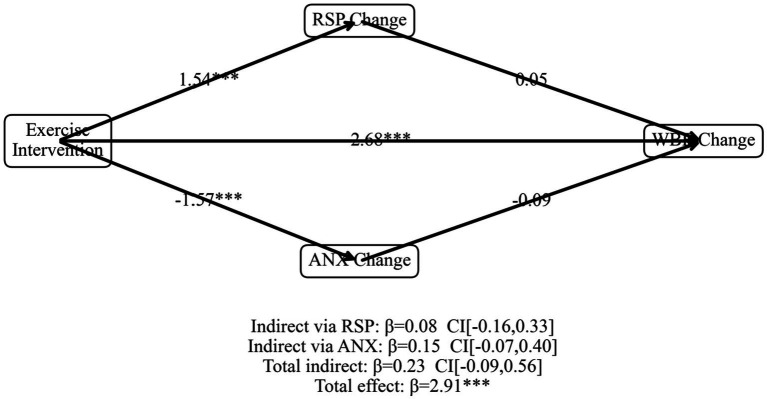
Parallel mediation model.

Table 7 presents the results of the parallel mediation analysis examining whether changes in romantic self-perception (RSP) and anxiety (ANX) mediated the relationship between the exercise intervention and changes in psychological wellbeing (WBE). All coefficients reported in the mediation model are unstandardized regression coefficients. The intervention significantly predicted changes in both mediators. Specifically, exercise participation significantly increased changes in RSP (*β* = 1.54, SE = 0.26, *p* < 0.001) and significantly decreased changes in ANX (*β* = −1.57, SE = 0.27, *p* < 0.001). However, neither mediator significantly predicted changes in psychological wellbeing. The effect of RSP change on WBE change was not statistically significant (*β* = 0.05, SE = 0.07, *p* = 0.470), and the effect of ANX change on WBE change was also not statistically significant (*β* = −0.09, SE = 0.07, *p* = 0.198). The direct effect of the exercise intervention on WBE change remained statistically significant (*β* = 2.68, SE = 0.33, *p* < 0.001). Bootstrap analyses based on 5,000 resamples indicated that the indirect effect through RSP was not statistically significant [*β* = 0.08, 95% CI (−0.16, 0.33)], and the indirect effect through ANX was also not statistically significant [*β* = 0.15, 95% CI (−0.07, 0.40)], as both confidence intervals included zero. The total indirect effect was likewise not statistically significant [*β* = 0.23, 95% CI (−0.09, 0.56)]. In contrast, the total effect of the intervention on WBE change remained statistically significant (*β* = 2.91, SE = 0.29, *p* < 0.001). Taken together, these findings indicate that although the exercise intervention significantly influenced both romantic self-perception and anxiety, neither variable significantly mediated the association between exercise participation and psychological wellbeing in the present model. Figure 2 illustrates the estimated paths in the parallel mediation model.

**Table 7:** Parallel mediation analysis results (bootstrap = 5,000).

Path	*β*	SE	*p*/95% CI	Sig.
Exercise → RSP change (a1)	1.54	0.26	< 0.001	***
Exercise → ANX change (a2)	−1.57	0.27	< 0.001	***
RSP change → WBE change (b1)	0.05	0.07	0.470	
ANX change → WBE change (b2)	−0.09	0.07	0.198	
Direct effect (c′)	2.68	0.33	< 0.001	***
Indirect effect via RSP	0.08	0.12	[−0.16, 0.33]	
Indirect effect via ANX	0.15	0.12	[−0.07, 0.40]	
Total indirect effect	0.23	0.16	[−0.09, 0.56]	
Total effect (c)	2.91	0.29	< 0.001	***

The indirect effects via romantic self-perception and anxiety were not significant because their bootstrap confidence intervals included zero.

***p < 0.001.

## 5 Discussion

### 5.1 Exercise intervention improves romantic self-perception and reduces anxiety

The findings of the present study support Hypotheses 1 and 2, indicating that participants who engaged in the structured exercise intervention showed greater improvement in romantic self-perception (RSP) and a larger reduction in anxiety (ANX) compared with those in the control group. The repeated-measures analyses revealed significant time effects and group × time interaction effects, suggesting that the psychological changes observed across the intervention period were associated with participation in the exercise program rather than with natural fluctuations over time, and although the control group exhibited minor changes between the pretest and posttest measurements, the magnitude of change was substantially larger in the intervention group, indicating that sustained engagement in physical activity contributed to more pronounced improvements in both relational self-evaluation and emotional regulation. These results are consistent with evidence showing that physical exercise can alleviate symptoms of depression and anxiety and may serve as a valuable complementary strategy for improving psychological health (Craft and Perna, 2004), and accumulating research further suggests that regular physical activity promotes emotional regulation, enhances self-esteem, and reduces psychological stress, thereby contributing to broader mental health benefits (Anderson and Shivakumar, 2013). Beyond these general effects, the present findings extend existing evidence by suggesting that the psychological benefits of exercise may reach into more domain-specific forms of self-evaluation, including how women perceive their attractiveness and relational worth in romantic contexts. A theoretically plausible explanation for this pattern draws on hierarchical models of self-concept, whereby repeated experiences of physical competence and bodily mastery during exercise progressively strengthen physical self-concept, and these improvements in physical self-evaluation generalize upward into broader self-evaluative domains, including romantic self-perception (Fox, 1997). As women develop greater confidence in their physical capabilities through sustained exercise participation, the appearance-related anxiety and self-monitoring that typically constrain romantic self-perception may be progressively reduced, allowing a more stable and positive sense of relational self-worth to emerge, a mechanism that is particularly relevant in the context of the present intervention given that the program was designed to maintain a supportive and non-competitive group environment, conditions that may have further facilitated positive bodily experiences and reduced evaluative threat during physical activity. The anxiety reduction observed in the intervention group is similarly consistent with both physiological and psychological accounts of exercise’s anxiolytic effects, as regular moderate-intensity aerobic exercise has been shown to modulate neurotransmitter systems, reduce inflammatory markers, and regulate hypothalamic–pituitary–adrenal axis activity in ways that collectively attenuate stress reactivity and reduce trait anxiety over time (Mikkelsen et al., 2017), while psychologically the repeated experience of successfully managing physical challenges during exercise may strengthen self-regulatory capacity and generalize to improved emotion regulation in non-exercise contexts, including interpersonal and social situations where anxiety is commonly experienced among female university students (Bernstein and McNally, 2018). In the specific cultural context of Chinese female university students, who are frequently subject to appearance-related social pressures and gender-role expectations that heighten evaluative anxiety in relational contexts, the anxiety-reducing effects of structured exercise participation may carry particular psychological significance by directly targeting one of the core mechanisms through which sociocultural pressures undermine romantic self-perception and emotional wellbeing. Taken together, the present findings suggest that participation in structured exercise may influence psychological functioning through both physical self-concept and emotional regulation pathways in ways that are theoretically coherent with existing models of exercise psychology, though these conclusions should be interpreted with appropriate caution given that the study was conducted within a specific cultural and institutional context and the generalizability of the findings to other populations and settings remains to be established through further research.

### 5.2 Exercise intervention enhances psychological wellbeing

The results of the present study support Hypothesis 3, indicating that participants who engaged in the exercise intervention demonstrated greater improvement in psychological wellbeing (WBE) compared with those in the control group, and the repeated-measures analyses revealed a significant time effect and a significant group × time interaction effect for psychological wellbeing, suggesting that the observed increase in wellbeing over the intervention period was associated with participation in the structured exercise program rather than with natural temporal variation. While the control group displayed relatively stable levels of wellbeing between the pretest and posttest measurements—with an effect size of *d* = 0.00 indicating a complete absence of change—participants in the intervention group experienced a meaningful increase in positive psychological functioning following regular participation in physical activity, with a within-group effect size of d = 0.93 indicating a large and practically significant improvement. These findings are consistent with prior research showing that higher levels of physical activity are associated with improvements in health-related quality of life and overall psychological wellbeing (Anokye et al., 2012), and evidence from large-scale reviews further indicates that engagement in physical activity is positively related to mental health outcomes including enhanced emotional wellbeing and reduced psychological distress (Biddle et al., 2019). Beyond these general associations, several features of the present intervention may help explain the magnitude of the wellbeing improvements observed. The group-based format of the exercise sessions provided participants with regular opportunities for positive social interaction and peer support within a structured and non-competitive environment, experiences that may have fulfilled basic psychological needs for relatedness and belonging as described within Self-Determination Theory (Ntoumanis et al., 2021). In addition, the twice-weekly schedule of supervised sessions created a consistent behavioral routine that may have contributed to psychological wellbeing through increased behavioral activation, reduced rumination, and the development of a sense of personal agency and accomplishment over the 8-week period. Research on lifestyle psychiatry similarly suggests that the wellbeing benefits of exercise extend beyond physiological mechanisms to encompass the social, behavioral, and identity-related dimensions of regular physical activity participation (Firth et al., 2020), and evidence that exercise supports psychological balance through improvements in self-regulatory capacity further suggests that the wellbeing gains observed in the present study may reflect the cumulative effect of repeated positive physical and social experiences rather than any single mechanism operating in isolation (Bernstein and McNally, 2018). Within the specific context of Chinese female university students, who frequently navigate competing demands related to academic performance, appearance-related social expectations, and interpersonal relationships, the structured exercise program may have provided a psychologically restorative context that offered temporary relief from evaluative pressures and a reliable source of positive affect and self-efficacy during a period of heightened developmental challenge. Taken together, the present findings suggest that participation in structured exercise programs may contribute to improvements in psychological wellbeing among female university students, though it should be noted that the present study assessed outcomes at a single posttest point immediately following the intervention, and whether these improvements are sustained over longer periods remains an open question that future longitudinal research should address.

### 5.3 Mediation effects of romantic self-perception and anxiety

Hypothesis 4 proposed that romantic self-perception (RSP) and anxiety (ANX) would mediate the relationship between the exercise intervention and psychological wellbeing (WBE). However, the results of the parallel mediation analysis did not support this hypothesis. Although the exercise intervention significantly predicted changes in both romantic self-perception and anxiety, and the total effect of exercise on psychological wellbeing remained statistically significant, the indirect effects through RSP and ANX were not significant because the bootstrap confidence intervals included zero, indicating that while exercise participation was associated with improvements in self-perception and reductions in anxiety, these variables did not statistically account for the relationship between exercise and psychological wellbeing within the current model. Several methodological and substantive explanations should be considered when interpreting this pattern of results. First, and most fundamentally, the present study employed only two measurement points—a pretest and an immediate posttest—which places an important constraint on the mediation analysis. Cross-sectional mediation models assessed at a single time point cannot establish the temporal precedence among variables that is required for causal mediation inference, and it is possible that the changes in RSP and ANX that occurred during the intervention period did not have sufficient time to translate into detectable improvements in psychological wellbeing by the posttest assessment, or that the sequencing of these changes did not conform to the simultaneous measurement design imposed by the study (Maxwell and Cole, 2007). Future studies employing three or more measurement points and latent growth curve modeling would be substantially better positioned to capture the dynamic unfolding of mediation processes over time. Second, the relatively homogeneous composition of the sample—female undergraduate students drawn from a restricted age range and institutional context in eastern China—may have compressed the variance in RSP and ANX change scores, thereby reducing the statistical power available to detect significant indirect effects even if theoretically meaningful mediation processes were operating. Third, the RSP scale used in the present study was newly developed, and although it demonstrated satisfactory psychometric properties, it is possible that the scale did not capture the full range of exercise-induced changes in romantic self-perception with sufficient sensitivity, particularly given that relational self-evaluations may shift gradually and subtly in response to bodily experience rather than producing the sharp pre-post changes that the current design was best suited to detect. Beyond these methodological considerations, substantive explanations for the non-significant mediation are also plausible. The psychological benefits of exercise may operate through multiple concurrent pathways simultaneously rather than through a sequential cognitive chain, and improvements in wellbeing may emerge directly from the broader experiential context of exercise participation—including changes in mood, increased behavioral activation, enhanced vitality, and the social rewards of group-based physical activity—without requiring a mediating pathway through specific psychological constructs such as romantic self-perception or anxiety reduction (Mikkelsen et al., 2017; Kandola et al., 2019). From this perspective, the absence of significant indirect effects does not indicate that RSP and ANX are theoretically irrelevant, but rather suggests that they represent two components within a more complex and multidimensional set of psychological processes through which physical activity influences wellbeing, processes that the present two-mediator model was not sufficiently comprehensive to fully capture. Taken together, these findings indicate that the relationship between exercise participation and psychological wellbeing in the present study operated primarily through a direct effect, and the proposed mediational pathways should be regarded as exploratory theoretical propositions requiring further empirical examination rather than as conclusive evidence of mechanism. Future research would benefit from adopting longitudinal designs with multiple assessment waves, incorporating additional candidate mediators such as body appreciation, self-compassion, and social belonging, and testing these pathways in more diverse samples to build a more complete understanding of how structured physical activity produces its psychological benefits among female university students.

### 5.4 Implications for university physical activity and mental health programs

The findings of the present study carry several implications for university-based health promotion and mental health support programs, though these implications should be understood as tentative suggestions grounded in a single eight-week intervention rather than as definitive prescriptions for practice. First, the results suggest that structured exercise interventions may contribute to improvements in romantic self-perception, reductions in anxiety, and increases in psychological wellbeing among female university students, outcomes that indicate physical activity should not be viewed solely as a strategy for improving physical health but also as a potentially important behavioral resource for supporting psychological adjustment during the transition to adulthood. Within university settings, where students frequently encounter academic pressure, social expectations, and identity-related challenges, opportunities for regular exercise may play a role in promoting emotional balance and psychological resilience, consistent with broader evidence linking physical activity to mental health and emotional functioning across diverse populations (Biddle et al., 2019). Second**, the present findings highlight the potential value of integrating physical activity into broader mental health promotion strategies in higher education. Traditional student support systems often focus primarily on counseling or cognitive-based interventions, whereas the present findings suggest that behavioral approaches such as exercise programs may provide a complementary pathway for improving emotional wellbeing. Participation in organized physical activity may create environments in which students can develop confidence, experience social interaction, and regulate emotional stress through active engagement rather than passive coping strategies, and research on lifestyle psychiatry similarly suggests that behavioral factors such as physical activity may play an important role in the prevention and management of mental health** difficulties (Firth et al., 2020). Third, the **present study points to the potential value of designing exercise programs that are accessible and supportive specifically for female students, and this consideration may be particularly relevant in Chinese university contexts where appearance-related social pressures, gender-role expectations, and culturally specific ideals of femininity may shape how women engage with and benefit from physical activity. Structured exercise environments that emphasize non-competitive participation, peer support, and bodily competence rather than appearance-based performance may be especially well suited to addressing the psychological needs of female students in these contexts, as such features are likely to reduce evaluative threat and facilitate the kind of positive physical self-experience that the theoretical framework of the present study identifies as central to improvements in romantic self-perception and psychological wellbeing. By designing exercise programs with these cultural and gender-specific considerations in mind, universities in China and similar East Asian institutional contexts may be better positioned to support female students in building healthier behavioral habits that contribute to psychological adjustment during the university years. Overall, the present findings suggest that structured physical activity programs warrant consideration as part of university health promotion initiatives, with the caveat that further research employing longer follow-up periods, active control conditions, and more diverse samples will be necessary before firm conclusions about the effectiveness and generalizability of exercise-based psychological interventions can be drawn.**

## 6 Limitation and conclusion

Several limitations should be acknowledged when interpreting the findings of the present study. First, although the study adopted a randomized controlled trial design, the sample was limited to female university students from a specific educational context in China, which may restrict the generalizability of the findings to other age groups, male students, or populations from different cultural and institutional settings. Second, all psychological outcomes were measured using self-report questionnaires, which may be influenced by response bias, social desirability, or temporary affective states. Furthermore, all study variables were assessed using self-report measures collected at the same two time points, which introduces the risk of common method bias. This shared method variance may have artificially inflated the observed associations among variables and should be considered when interpreting the pattern of correlations and the results of the mediation analysis in particular. Future research should consider supplementing self-report measures with objective behavioral or physiological indicators to reduce this source of bias. Third, the intervention effects were assessed only at pretest and immediate posttest, and no long-term follow-up data were collected; therefore, the durability of the observed improvements remains unclear. Fourth, although the mediation model tested romantic self-perception and anxiety as potential explanatory mechanisms, the indirect effects were not statistically significant, suggesting that other unmeasured psychological, social, or behavioral processes may also contribute to the relationship between exercise intervention and psychological wellbeing. In addition, because only two measurement points were available in the present study, the mediation analysis should be interpreted as exploratory rather than definitive evidence of causal mechanisms, as cross-sectional mediation designs cannot establish the temporal precedence among variables that is required for causal inference. Fifth, the present study employed a passive control condition rather than an active control group, which limits the extent to which the observed improvements in the intervention group can be attributed specifically to the physical exercise component. The absence of an active control condition means that non-specific factors, including the Hawthorne effect, the psychological benefits of structured routine participation, and the social interaction inherent in group-based exercise sessions, cannot be ruled out as contributors to the observed outcomes. Future research should incorporate an active control condition matched for group contact time to isolate the specific contribution of physical exercise to the psychological changes observed. Sixth, because the intervention focused on a structured exercise program delivered in a supportive group setting, the findings should be interpreted as reflecting the overall effect of participation in the intervention context rather than the isolated effect of any single exercise component. Finally, the study was not preregistered, which should be considered when interpreting the transparency and confirmatory status of the analyses.

Despite these limitations, the present study examined the effects of a structured exercise intervention on romantic self-perception, anxiety, and psychological wellbeing among female university students and yielded several important findings. Participants who engaged in the exercise program showed greater improvement in romantic self-perception and psychological wellbeing, as well as a greater reduction in anxiety, compared with those in the control group. Repeated-measures analyses revealed significant time effects and group × time interaction effects for the key psychological outcomes, suggesting that the observed improvements were associated with participation in the exercise intervention rather than with general temporal variation. These findings support Hypotheses 1, 2, and 3 and provide preliminary evidence that regular physical activity may positively influence both emotional regulation and self-related evaluations among female university students within the specific cultural and institutional context examined. At the same time, the mediation analysis did not support Hypothesis 4. Although the exercise intervention significantly predicted changes in romantic self-perception and anxiety, the indirect effects through these variables were not statistically significant because the bootstrap confidence intervals included zero. This result should be interpreted with caution given the methodological constraints of the present design, particularly the use of only two measurement points, which limits the capacity of the mediation model to establish temporal precedence among variables and precludes causal mediation inference. The proposed mediational pathways should therefore be regarded as exploratory theoretical propositions that warrant further examination in longitudinal designs with multiple assessment waves rather than as established evidence of psychological mechanism. The findings indicate that the association between exercise participation and psychological wellbeing in the present study was primarily characterized by a direct effect, and that the psychological benefits of exercise likely operate through multiple mechanisms that extend beyond the variables examined in the current analysis. Overall, the results provide preliminary support for the potential role of structured physical activity as a behavioral strategy for promoting psychological wellbeing among female university students in Chinese higher education settings. By providing opportunities for regular behavioral engagement, emotional regulation, and positive self-evaluation within a supportive group context, structured exercise programs may help students manage stress, strengthen self-confidence, and maintain psychological stability during the university years, though the generalizability of these conclusions to other cultural contexts, student populations, and exercise formats remains to be established through further research.

## Data Availability

The raw data supporting the conclusions of this article will be made available by the authors, without undue reservation.

## References

[ref1] Anderson E. Shivakumar G. (2013). Effects of exercise and physical activity on anxiety. Front. Psych. 4:27. doi: 10.3389/fpsyt.2013.00027, 23630504 PMC3632802

[ref2] Anokye N. K. Trueman P. Green C. Pavey T. G. Taylor R. S. (2012). Physical activity and health related quality of life. BMC Public Health 12:624. doi: 10.1186/1471-2458-12-624, 22871153 PMC3490805

[ref3] Avalos L. Tylka T. L. Wood Barcalow N. (2005). The body appreciation scale: development and psychometric evaluation. Body Image 2, 285–297. doi: 10.1016/j.bodyim.2005.06.002 18089195

[ref4] Barelds D. P. (2005). Self and partner personality in intimate relationships. Eur. J. Personal. 19, 501–518. doi: 10.1002/per.549

[ref5] Bartley C. A. Hay M. Bloch M. H. (2013). Meta analysis: aerobic exercise for the treatment of anxiety disorders. Prog. Neuro-Psychopharmacol. Biol. Psychiatry 45, 34–39. doi: 10.1016/j.pnpbp.2013.04.016, 23643675

[ref6] Bernstein E. E. McNally R. J. (2018). Exercise as a buffer against difficulties with emotion regulation: a pathway to emotional wellbeing. Behav. Res. Ther. 109, 29–36. doi: 10.1016/j.brat.2018.07.010, 30081242

[ref7] Biddle S. J. H. Ciaccioni S. Thomas G. Vergeer I. (2019). Physical activity and mental health in children and adolescents: an updated review of reviews and an analysis of causality. Psychol. Sport Exerc. 42, 146–155. doi: 10.1016/j.psychsport.2018.08.011

[ref9003] Brislin R. W. (1970). Back-Translation for Cross-Cultural Research. J Cross-Cultural Psychol. 1, 185–216.

[ref8] Carraça E. V. Silva M. N. Markland D. Vieira P. N. Minderico C. S. Sardinha L. B. . (2011). Body image change and improved eating self-regulation in a weight management intervention in women. Int. J. Behav. Nutr. Phys. Act. 8:75. doi: 10.1186/1479-5868-8-75, 21767360 PMC3150233

[ref9] Cohen J. (1988). Statistical Power Analysis for the Behavioral Sciences. 2nd Edn. Hillsdale, NJ: Lawrence Erlbaum Associates.

[ref10] Cox A. E. Ullrich French S. Sabiston C. M. (2013). Using motivation regulations in a person centered approach to examine the link between social physique anxiety in physical education and physical activity related outcomes in adolescents. Psychol. Sport Exerc. 14, 461–467. doi: 10.1016/j.psychsport.2013.01.005

[ref11] Craft L. L. Perna F. M. (2004). The benefits of exercise for the clinically depressed. Prim. Care Companion J. Clin. Psychiatry 6, 104–111. doi: 10.4088/pcc.v06n0301, 15361924 PMC474733

[ref12] Deslandes A. Moraes H. Ferreira C. Veiga H. Silveira H. Mouta R. . (2009). Exercise and mental health: many reasons to move. Neuropsychobiology 59, 191–198. doi: 10.1159/000223730, 19521110

[ref13] Duan X. Wang X. Li X. Li S. Zhong Y. Bu T. (2022). Effect of mass sports activity on prosocial behavior: a sequential mediation model of flow trait and subjective wellbeing. Front. Public Health 10:960870. doi: 10.3389/fpubh.2022.960870, 35979458 PMC9376381

[ref14] El Oirdi H. Boujdi R. El Kabbaoui M. Eloirdi A. Akhittouch A. El Kharrim K.. (2024). Effect of exercise intensity on sleep quality among school adolescents in the provincial Directorate of Meknes-Morocco: sports and health. E3S Web of Conferences, 527, 04001.

[ref15] El Oirdi H. Bouziani A. El Oirdi A. Mostyafi J. Hamrani A. El Kharrim K. . (2021). Physical inactivity, sedentary habits and eating habits among Moroccan adolescents. E3S Web of Conferences, 319, 01022.

[ref16] El Oirdi H. Eloirdi A. Boulebatt S. El Kharrim K. Belghity D.. (2023). Correlational relationship between physical activity and sedentary behavior among Moroccan high school students. SHS Web of Conferences, 175, 01044.

[ref17] Firth J. Solmi M. Wootton R. E. Vancampfort D. Schuch F. B. Hoare E. . (2020). A meta review of “lifestyle psychiatry”: the role of exercise, smoking, diet and sleep in the prevention and treatment of mental disorders. World Psychiatry 19, 360–380. doi: 10.1002/wps.20773, 32931092 PMC7491615

[ref18] Fox K. R. (1997). “The physical self and processes in self-esteem development,” in The Physical Self: From Motivation to Well-Being, ed. Fox K. R. (Champaign, IL: Human Kinetics), 111–139.

[ref19] Gan L. Jiang Y. (2024). How is physical activity associated with social anxiety among college students? The mediating role of body image and the moderating role of self-esteem. Curr. Psychol. 43, 34679–34687. doi: 10.1007/s12144-024-06920-7

[ref20] Hausenblas H. A. Fallon E. A. (2006). Exercise and body image: a meta analysis. Psychol. Health 21, 33–47. doi: 10.1080/14768320500105270

[ref21] Homan K. J. Tylka T. L. (2018). Development and exploration of the gratitude model of body appreciation. Body Image 25, 14–22. doi: 10.1016/j.bodyim.2018.01.008, 29428332

[ref22] Impett E. A. Schooler D. Tolman D. L. (2006). To be seen and not heard: femininity ideology and adolescent girls’ sexual health. Arch. Sex. Behav. 35, 129–142. doi: 10.1007/s10508-005-9016-0, 16752117

[ref23] Kandola A. Ashdown Franks G. Hendrikse J. Sabiston C. M. Stubbs B. (2019). Physical activity and depression: towards understanding the antidepressant mechanisms of physical activity. Neurosci. Biobehav. Rev. 107, 525–539. doi: 10.1016/j.neubiorev.2019.09.040, 31586447

[ref24] Kaur N. Kaur P. Kumar S. (2025). Physiological impact of structured physical education programs on athletic performance and well being in youth sports. Int. J. Res. Publ. Rev. 6, 4279–4281. doi: 10.55248/gengpi.6.0425.1462

[ref25] Krane V. Choi P. Y. L. Baird S. M. Aimar C. M. Kauer K. J. (2004). Living the paradox: female athletes negotiate femininity and muscularity. Sex Roles 50, 315–329. doi: 10.1023/B:SERS.0000018888.48437.4f

[ref26] Maxwell S. E. Cole D. A. (2007). Bias in cross-sectional analyses of longitudinal mediation. Psychol. Methods 12, 23–44. doi: 10.1037/1082-989X.12.1.23 17402810

[ref27] Meltzer A. L. McNulty J. K. (2014). “Tell me I’m sexy… and otherwise valuable”: body valuation and relationship satisfaction. Pers. Relat. 21, 68–87. doi: 10.1111/pere.12018, 24683309 PMC3964620

[ref28] Meltzer A. L. McNulty J. K. Jackson G. L. Karney B. R. (2014). Sex differences in the implications of partner physical attractiveness for the trajectory of marital satisfaction. J. Pers. Soc. Psychol. 106, 418–428. doi: 10.1037/a0034424, 24128188 PMC4011637

[ref29] Mikkelsen K. Stojanovska L. Polenakovic M. Bosevski M. Apostolopoulos V. (2017). Exercise and mental health. Maturitas 106, 48–56. doi: 10.1016/j.maturitas.2017.09.003, 29150166

[ref30] Murray S. L. Holmes J. G. Griffin D. W. (1996). The benefits of positive illusions: idealization and the construction of satisfaction in close relationships. J. Pers. Soc. Psychol. 70, 79–98. doi: 10.1037/0022-3514.70.1.79

[ref31] Neville R. D. Draper C. E. Cooper T. J. Abdullah M. M. Lakes K. D. (2021). Association between engagement in physical activity and adaptive behavior in young children with autism Spectrum disorder. Ment. Health Phys. Act. 20:100389. doi: 10.1016/j.mhpa.2021.100389

[ref32] Nolen-Hoeksema S. (2012). Emotion regulation and psychopathology: the role of gender. Annu. Rev. Clin. Psychol. 8, 161–187. doi: 10.1146/annurev-clinpsy-032511-143109, 22035243

[ref33] Ntoumanis N. Ng J. Y. Y. Prestwich A. Quested E. Hancox J. E. Thøgersen-Ntoumani C. . (2021). A meta-analysis of self-determination theory-informed intervention studies in the health domain: the importance of motivation and technique. Health Psychol. Rev. 15, 214–244. doi: 10.1080/17437199.2020.1718529, 31983293

[ref34] Rao U. T. Noronha J. A. Adiga K. (2020). Effect of aerobic exercises on depressive symptoms, anxiety, self esteem, and quality of life among adults with depression. Clin. Epidemiol. Glob. Health 8, 1147–1151. doi: 10.1016/j.cegh.2020.04.006

[ref9002] Ryff C. D. Keyes C. L. (1995). The structure of psychological well-being revisited. J Person Soc Psychol. 69, 719–727. doi: 10.1037//0022-3514.69.4.719, 7473027

[ref35] Sabiston C. M. Pila E. Vani M. Thogersen Ntoumani C. (2019). Body image, physical activity, and sport: a scoping review. Psychol. Sport Exerc. 42, 48–57. doi: 10.1016/j.psychsport.2018.12.010

[ref36] Salmon P. (2001). Effects of physical exercise on anxiety, depression, and sensitivity to stress: a unifying theory. Clin. Psychol. Rev. 21, 33–61. doi: 10.1016/S0272-7358(99)00032-X, 11148895

[ref37] Sanchez D. T. Kiefer A. K. (2007). Body concerns in and out of the bedroom: implications for sexual pleasure and problems. Arch. Sex. Behav. 36, 808–820. doi: 10.1007/s10508-007-9205-0, 17657464

[ref38] Schuch F. B. Stubbs B. Meyer J. Heissel A. Zech P. Vancampfort D. . (2019). Physical activity protects from incident anxiety: a meta analysis of prospective cohort studies. Depress. Anxiety 36, 846–858. doi: 10.1002/da.22915, 31209958

[ref39] Slater A. Tiggemann M. (2011). Gender differences in adolescent sport participation, teasing, self objectification and body image concerns. J. Adolesc. 34, 455–463. doi: 10.1016/j.adolescence.2010.06.007, 20643477

[ref9001] Spitzer R. L. Kroenke K. Williams J. B. Löwe R. L. (2006). A brief measure for assessing generalized anxiety disorder: the GAD-7. Archiv Intern Med. 166, 1092–1097. doi: 10.1001/archinte.166.10.1092, 16717171

[ref40] Stubbs B. Vancampfort D. Rosenbaum S. Firth J. Cosco T. Veronese N. . (2017). An examination of the anxiolytic effects of exercise for people with anxiety and stress related disorders: a meta analysis. Psychiatry Res. 249, 102–108. doi: 10.1016/j.psychres.2016.12.020, 28088704

[ref41] Swami V. Barron D. Furnham A. (2018). Exposure to natural environments, and photographs of natural environments, promotes more positive body image. Body Image 24, 82–94. doi: 10.1016/j.bodyim.2017.12.006, 29331662

[ref42] Tolman D. L. Impett E. A. Tracy A. J. Michael A. (2006). Looking good, sounding good: femininity ideology and adolescent girls’ mental health. Psychol. Women Q. 30, 85–95. doi: 10.1111/j.1471-6402.2006.00265.x

[ref43] Torres S. (2021). School based body image intervention: overcoming challenges to dissemination. J. Adolesc. Health 68, 229–230. doi: 10.1016/j.jadohealth.2020.11.002, 33541597

[ref44] Vandenbosch L. Eggermont S. (2012). Understanding sexual objectification: a comprehensive approach toward media exposure and girls’ internalization of beauty ideals, self-objectification, and body surveillance. J. Commun. 62, 869–887. doi: 10.1111/j.1460-2466.2012.01667.x

[ref45] Vankim N. A. Nelson T. F. (2013). Vigorous physical activity, mental health, perceived stress, and socializing among college students. Am. J. Health Promot. 28, 7–15. doi: 10.4278/ajhp.111101-QUAN-395, 23470187 PMC3758412

[ref46] Weinstein A. A. Koehmstedt C. Kop W. J. (2017). Mental health consequences of exercise withdrawal: a systematic review. Gen. Hosp. Psychiatry 49, 11–18. doi: 10.1016/j.genhosppsych.2017.06.001, 28625704

[ref47] Wipfli B. M. Rethorst C. D. Landers D. M. (2008). The anxiolytic effects of exercise: a meta analysis of randomized trials and dose response analysis. J. Sport Exerc. Psychol. 30, 392–410. doi: 10.1123/jsep.30.4.392, 18723899

[ref48] Zhang X. Y. Wu W. X. Shen L. P. Ji M. J. Zhao P. F. Yu L. . (2024). A role for the cerebellum in motor triggered alleviation of anxiety. Neuron 112, 1165–1181.e8. doi: 10.1016/j.neuron.2024.01.007, 38301648

